# Integrin α2 marks a niche of trophoblast progenitor cells in first trimester human placenta

**DOI:** 10.1242/dev.162305

**Published:** 2018-04-16

**Authors:** Cheryl Q. E. Lee, Margherita Y. Turco, Lucy Gardner, Benjamin D. Simons, Myriam Hemberger, Ashley Moffett

**Affiliations:** 1 Department of Pathology, Tennis Court Road, University of Cambridge, Cambridge CB2 1QP, UK; 2 Centre for Trophoblast Research, Tennis Court Road, University of Cambridge, Cambridge CB2 3DY, UK; 3 Cavendish Laboratory, Department of Physics, University of Cambridge, J.J. Thomson Avenue, Cambridge CB3 0HE, UK; 4 The Wellcome Trust/Cancer Research UK Gurdon Institute, University of Cambridge, Tennis Court Road, Cambridge CB2 1QN, UK; 5 Wellcome Trust-Medical Research Council Stem Cell Institute, University of Cambridge, Cambridge CB2 1QR, UK; 6 Epigenetics Programme, The Babraham Institute, Babraham Research Campus, Cambridge CB22 3AT, UK

**Keywords:** Placenta, Stem cells, Integrin α2, Progenitor, Lineage tracing

## Abstract

During pregnancy the trophoblast cells of the placenta are the only fetal cells in direct contact with maternal blood and decidua. Their functions include transport of nutrients and oxygen, secretion of pregnancy hormones, remodelling of the uterine arteries, and communicating with maternal cells. Despite the importance of trophoblast cells in placental development and successful pregnancy, little is known about the identity, location and differentiation of human trophoblast progenitors. We identify a proliferative trophoblast niche at the base of the cytotrophoblast cell columns in first trimester placentas that is characterised by integrin α2 (ITGA2) expression. Pulse-chase experiments with 5-iodo-2′-deoxyuridine indicate that these cells might contribute to both villous (VCT) and extravillous (EVT) lineages. These proliferating trophoblast cells can be isolated by flow cytometry using ITGA2 as a marker and express genes from both VCT and EVT. Microarray expression analysis shows that ITAG2^+^ cells display a unique transcriptional signature, including genes involved in NOTCH signalling, and exhibit a combination of epithelial and mesenchymal characteristics. ITGA2 thus marks a niche allowing the study of pure populations of trophoblast progenitor cells.

## Abstract (precis)

**Summary:** ITGA2 marks a proliferative trophoblast progenitor compartment in first trimester human placenta that appears to be regulated by NOTCH signalling and exhibits a unique combination of epithelial and mesenchymal expression characteristics.

## INTRODUCTION

Despite the rapid growth of the human placenta in the early weeks of gestation, little is known about the identity and location of a proliferative or even self-renewing niche of trophoblast stem or progenitor cells. By definition, stem cells are cells capable of unlimited self-renewal and differentiation into several lineages. Murine trophoblast stem cells (TSCs) can self-renew in culture and contribute to all the trophoblast lineages *in vivo* (Tanaka et al., 1998). Because the existence of putative TSCs in the human placenta is unknown, we refer to the proliferative cells in human placentas as trophoblast progenitors (TPs). Trophoblast differentiates along two main pathways, villous and extravillous. In the first trimester, the placental villus consists of a stromal core covered by two layers of trophoblast: an inner layer of villous cytotrophoblast (VCT) from which cells differentiate and fuse to form an outer layer of syncytiotrophoblast (ST). Extravillous cytotrophoblast (EVT) cells push through the ST in places forming cytotrophoblast cell columns (CCCs) from where EVT can invade into the maternal decidua. Other villi float freely in maternal blood in the intervillous space, the site of maternal/fetal transfer of nutrients and gases.

Stem cells in other tissues are often characterised by expression of specific types of integrins; for example, integrin β1 demarcates stem cells in epithelia and mammary glands (Jensen et al., 1999; Jones and Watt, 1993; Shackleton et al., 2006; Stingl et al., 2006; Taddei et al., 2008). Integrins have important functional roles in these cells, as alteration of integrin levels can affect proliferation and differentiation, either by direct signalling or indirectly by anchoring the cells in a specific niche (Ellis and Tanentzapf, 2010; Hirsch et al., 2002; Sastry et al., 1996). Therefore, integrins are good candidates to identify proliferative trophoblast and to isolate live cells.

To characterise human TPs, we first confirmed the location of putative TP niches by staining first trimester placentas for proliferative markers (Arnholdt et al., 1991; Bulmer et al., 1988; Chan et al., 1999; Enders, 1968; Mühlhauser et al., 1993; Vićovac et al., 1995). We focussed on first trimester placentas as there is a negative correlation between gestational age and the proportion of proliferative placental cells (Arnholdt et al., 1991; Hemberger et al., 2010; Horii et al., 2016). We found a surface protein, integrin α2 (ITGA2), expressed on the proliferative trophoblast cells at the base of the CCCs, enabling us to isolate and characterise human TPs using flow cytometry. The gene expression profile of these cells reveals that they are enriched in NOTCH signalling pathways and unusual mesenchymal-like characteristics. Using thymidine analogues, we were able to pulse chase these cells and show that they might contribute to both VCT and EVT. Our findings confirm previous reports that suggested the existence of a TP niche at the base of the CCCs (Mühlhauser et al., 1993; Vićovac et al., 1995).

## RESULTS

### Location of proliferating trophoblast cells in the first trimester

**Fig. 1.**
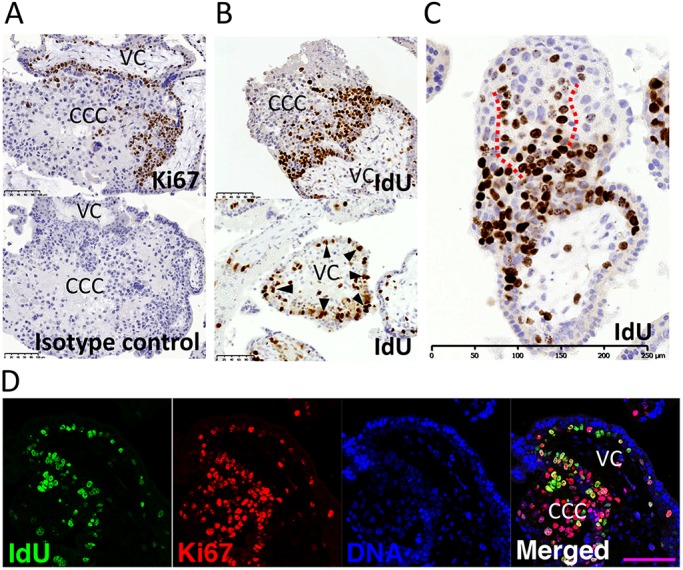
**Proliferative trophoblast cells mostly reside at the base of the CCCs.** (A,B) Placental sections stained for Ki67 (A; *n*=6) and IdU (B; *n*=10). Arrowheads point to IdU^+^ cells within villi that are highly proliferative. (C) Example of strings of IdU^+^ cells in the CCCs. Dashed lines demarcate strings of consecutively labelled cells, suggesting that neighbouring cells have synchronised cell cycles and are perhaps recent relatives. (D) Co-immunostaining for Ki67 and IdU (*n*=3). VC, villous core. Scale bars: 100 μm (A,B,D); 250 μm (C).

To identify the location of TPs in first trimester placentas, we first stained for proliferative cells using Ki67 (MKI67), which is expressed in the entire cell cycle except G_0_ phase, and 5-iodo-2′-deoxyuridine (IdU), a base analogue incorporated during S phase (Gerdes et al., 1984). Fresh placental explants were incubated in IdU for an hour and fixed immediately for immunohistochemistry. In the villous placenta, no Ki67-positive cells are ever seen in ST and staining in VCT is patchy [Fig. 1A; *n*=6, gestational age (G.A.)=7-12 weeks]. In the CCCs, Ki67 staining is restricted to the base region. Similar findings were made for IdU with most cells in S phase seen at the base of CCCs and in adjacent VCT cells (Fig. 1B; *n*=10, G.A.=7-12 weeks). In the CCCs, the IdU^+^ trophoblast cells are aligned to form strings of consecutively labelled cells, suggesting that neighbouring cells have synchronised cell cycles and are probably recent relatives (Fig. 1C). In VCT, patches of cells in S phase are occasionally observed but these are rare and most villi contain only a few IdU^+^ VCT (Fig. 1B, arrowheads). Co-staining for IdU and Ki67 shows that IdU^+^ cells are concentrated in a smaller region at the base of the CCCs, whereas Ki67 staining extends for more cell layers distally (Fig. 1D; *n*=3, G.A.=8-11 weeks). This indicates that cells in the CCCs lose their proliferative potential as they move further away from the villus and that the proximal CCC is the most likely location for TPs.

### ITGA2 marks proliferative cells at the base of the CCC

**Fig. 2.**
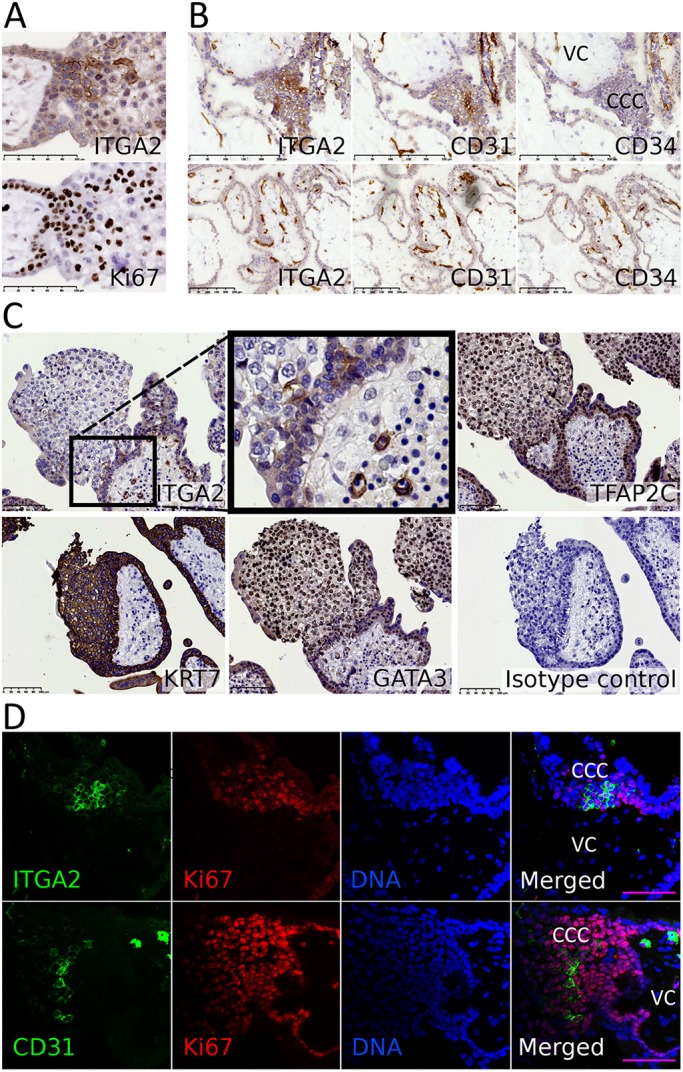
**Proliferative trophoblast cells mostly reside at the base of the CCCs.** (A) Serial sections show that ITGA2 is expressed on Ki67^+^ cells (*n*=3). (B) Placental serial sections stained for ITGA2, CD31 and CD34 (*n*=3). Only CD31 and ITGA2 are present on proximal CCCs (top) whereas all three proteins are expressed on endothelial cells (bottom). (C) Serial sections show that ITGA2^+^ trophoblast cells are positive for Ki67, TFAP2C, GATA3 and KRT7. (D) Co-immunostaining for CD31 and ITGA2 with Ki67 (*n*=3). VC, villous core. Scale bars: 100 μm (A,C,D); 200 μm (B, top); 250 μm (B, bottom).

To search for a surface marker that could be used to isolate putative TPs, we studied integrin expression in the proliferative niche at the base of the CCCs, and found that ITGA2 is expressed specifically in trophoblast cells in this location (Fig. 2A; *n*=3, G.A.=8-9 weeks). Endothelial cells in the villous core are also ITGA2^+^, seen by staining of serial sections with CD31 (PECAM1; Fig. 2B). In addition, a smaller cluster of CD31^+^ cells is present at the base of the CCCs (Fig. 2B; *n*=3, G.A.=8-10 weeks). We therefore stained serial sections for another endothelial marker, CD34, to ensure that ITGA2^+^ and CD31^+^ cells at the base of the CCCs are not endothelial cells. CD34, CD31 and ITGA2 colocalise on endothelial cells but only CD31 and ITGA2 are present at the base of the CCCs (Fig. 2B; *n*=3, G.A.=8-10 weeks). As further proof that the ITGA2^+^ cells are trophoblast, serial sections were stained for the epithelial marker cytokeratin 7 (KRT7) and transcription factors (TFs) characteristic of trophoblast, GATA3 and TFAP2C (Lee et al., 2016). The ITGA2^+^ cells at the base of the CCCs express these TFs and KRT7, confirming that they are trophoblast (Fig. 2C; *n*=3, G.A.=8-9 weeks).

As ITGA2 and CD31 are expressed in a region with high proliferative activity, we co-stained ITGA2 and CD31 with Ki67. The majority of ITGA2^+^ and CD31^+^ trophoblast cells are Ki67^+^ and these cells cluster near the basement membrane at the base of the CCCs, surrounded by Ki67^+^/ITGA2^−^/CD31^−^ cells (Fig. 2D; *n*=3, G.A.=7-8 weeks). These findings show that CD31 and ITGA2 mark a niche of proliferative trophoblast cells.

### Characterisation of isolated ITGA2^+^ cells

**Fig. 3.**
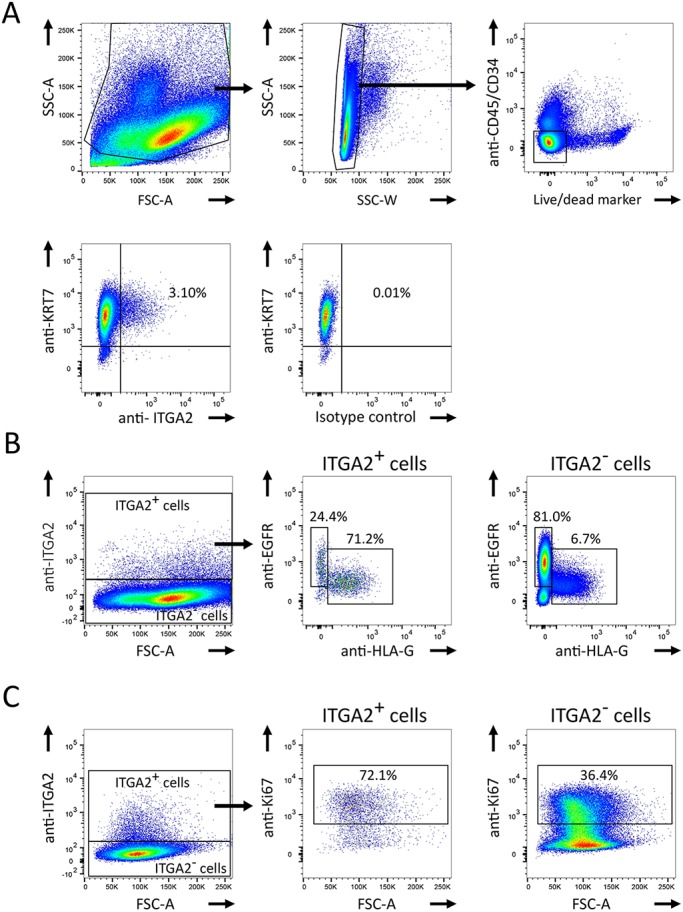
**ITGA2 can be used to characterise and isolate TPs by flow cytometry.** (A) After gating out debris, cell doublets, CD45^+^ leukocytes and CD34^+^ endothelial cells, a proportion of KRT7^+^ trophoblast cells are ITGA2^+^ in the remaining fraction (3.60±0.74%; mean±s.e.m.; *n*=3). (B) Expression of EGFR (VCT) and HLA-G (EVT) on ITGA2^+^ (middle panel) and ITGA2^−^ (right panel) cells (*n*=3). Gating of ITGA2^+^ cells is shown in the left panel. (C) Ki67^+^ cells on cell populations gated as in B into ITGA2^+^ (middle panel) and ITGA2^−^ (right panel) cells (71.3±0.3% versus 42.5±4.3%; mean±s.e.m.; *n*=3). Figures show percentage values for the sample presented.

To isolate these cells by flow cytometry, we used an antibody to ITGA2 because CD31 is sensitive to the enzymatic digestion performed as part of our isolation procedure. Single, live cells were gated, and leukocytes and endothelial cells were excluded based on CD45 (PTPRC) and CD34 expression, respectively (Fig. 3A). About 3.60±0.74% (mean±s.e.m.) of cells in the remaining fraction are ITGA2^+^ (Fig. 3A; *n*=3, G.A.=7-8 weeks). These ITGA2^+^ cells are all KRT7^+^ and contain both HLA-G^+^ and EGFR^+^ cells demarcating EVT and VCT, respectively (Fig. 3A,B; *n*=3, G.A.=7-8 weeks) (Apps et al., 2011). We further determined that the percentage of Ki67^+^ cells is much higher in the ITGA2^+^ population than in the ITGA2^−^ fraction [Fig. 3C; 71.3±0.3% versus 42.5±4.3% (mean±s.e.m.); *n*=3, G.A.=7-8 weeks].

To provide further confirmation of the trophoblast identity of ITGA2^+^ cells, we investigated the methylation status of the *ELF5* promoter. The *ELF5* promoter is hypomethylated in human and mouse trophoblast cells compared with cells that originate from the embryonic lineage, which includes mesenchymal cells of the villous core and vascular endothelial cells (Lee et al., 2016; Ng et al., 2008). We compared the *ELF5* promoter by bisulphite sequencing in three trophoblast populations: ITGA2^+^ cells, VCT and EVT. ITGA2^+^ cells were isolated by flow cytometry first, followed by VCT and EVT from the remaining cells using EGFR and HLA-G, respectively (termed A-E-G populations). The *ELF5* promoter is hypomethylated in all three populations, with no indication that *ELF5* is differentially methylated in proliferative or differentiated trophoblast (Fig. S1; *n*=8 independent clones per donor from two donors). We have shown in a previous publication that the *ELF5* promoter is hypermethylated in placental mesenchymal cells (Lee et al., 2016). Similar findings for other non-trophoblast cells have been reported recently (Okae et al., 2018). The hypomethylation of the *ELF5* promoter in ITGA2^+^ cells therefore provides additional proof of the trophoblast identity of our isolated ITGA2^+^ cells.

These findings confirm that ITGA2 can be used as a marker to isolate a unique subpopulation of trophoblast cells from the proliferative niche at the base of the CCCs.

### Gene expression profile of ITGA2^+^ trophoblast

**Fig. 4.**
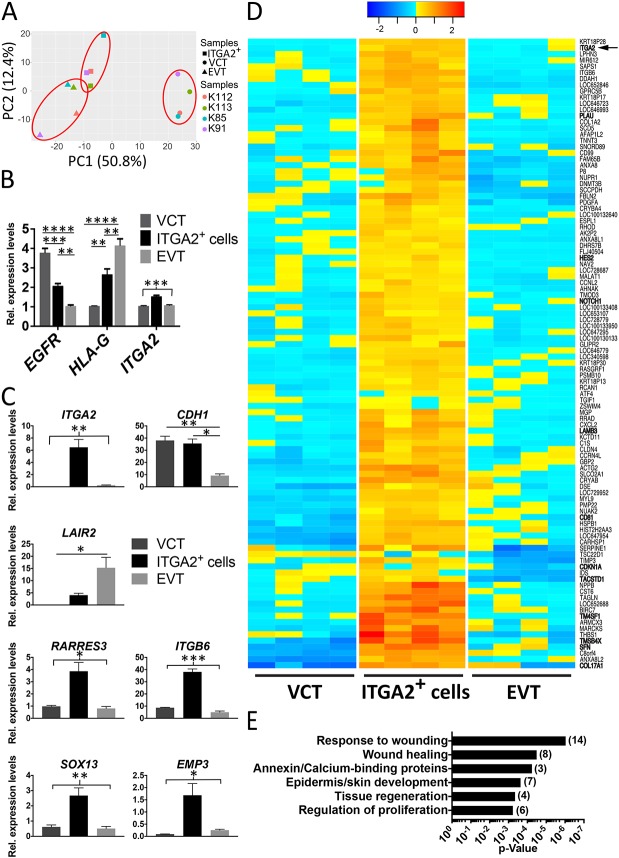
**ITGA2^+^ trophoblast cells have a unique gene expression profile compared with EVT and VCT.** (A) Principal component (PC) analysis is based on 23,196 genes with coefficient of variation>0.1. The analysis shows that ITGA2^+^ cells, VCT and EVT isolated from four different donors separate into distinct clusters. (B) *EGFR*, *HLA-G* and *ITGA2* are upregulated in their corresponding cell populations on the microarray. (C) RT-qPCR validation of some candidate genes as indicated by the microarray results. *P<0.05; **P<0.01; ***P<0.005; ****P<0.0001; one-way ANOVA followed by Tukey's multiple comparisons test. (D) Heat map of genes upregulated specifically in ITGA2^+^ trophoblast cells. Genes that we have mentioned in the text are emphasised in bold. (E) Functional clusters that are enriched in ITGA2^+^ cells, based on gene ontology analysis using DAVID. Number of genes associated with each process is written in parentheses.

To understand how cells located in the proximal CCC differ from those in the distal CCC and from VCT, the transcriptomes of the A-E-G populations from four placentas (G.A.=8-9 weeks) were compared by microarray. The samples cluster according to cell type by principal component analysis (PCA), suggesting that there is high purity of the three populations with little difference between individual donors (Fig. 4A). Hierarchical clustering provides further support that the ITGA2^+^ cells are a distinct population from EVT and VCT (Fig. S2A). Genes differentially expressed between VCT and EVT are similar to our previous findings (Fig. S2B) (Apps et al., 2011). Levels for *ITGA2*, *EGFR* and *HLA-G* are also the highest in the ITGA2^+^ cells, VCT and EVT, respectively, further verifying the identity and purity of the isolated cell types (Fig. 4B). The microarray results were validated on several candidate genes by RT-qPCR (Fig. 4C).

As the RT-qPCR results showed that a 1.23-fold difference in gene expression on the microarray was significant, we used a false discovery rate (FDR) of <0.05 and 1.23-fold difference in gene expression and found that 102 genes are more highly expressed in ITGA2^+^ cells than the other two trophoblast populations (Table S1, Fig. 4D). Using these genes for gene ontology analysis identified the terms wound healing, tissue regeneration and proliferation amongst the categories with strongest significance and enrichment (Fig. 4E). There are far fewer genes downregulated in ITGA2^+^ cells compared with the other two cell populations (Fig. S2C, Table S2).

**Fig. 5.**
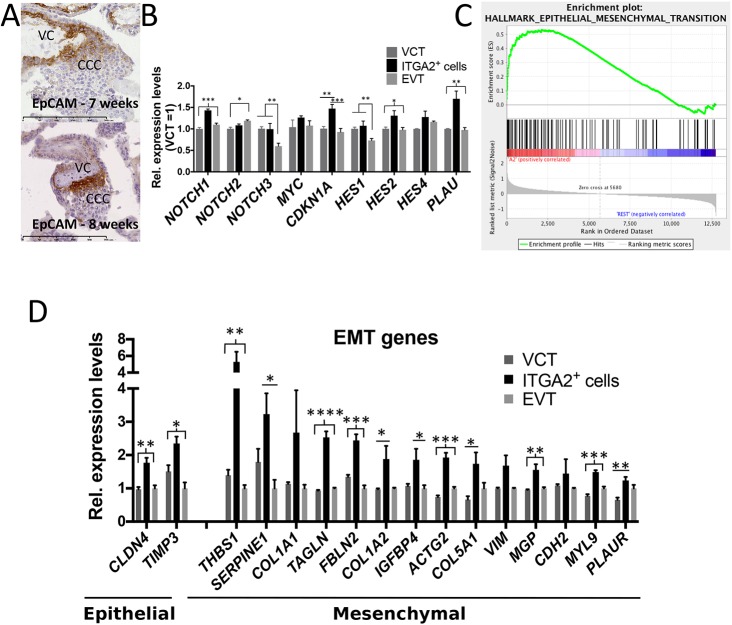
**ITGA2^+^ trophoblast cells are enriched in NOTCH signalling components and exhibit distinct EMT marker expression.** (A) EpCAM becomes restricted from VCT to the base of the CCCs between 7 and 8 weeks (*n*=3 for 6-7 weeks and for 8-10 weeks). VC, villous core. (B) Microarray data of expression of NOTCH pathway components. *NOTCH4* and other HES genes were not detectable on the microarray. (C) GSEA shows that the EMT pathway is activated in ITGA2^+^ trophoblast. (D) A specific subset of EMT genes, indicative of a mixed epithelial and mesenchymal identity, is upregulated in ITGA2^+^ trophoblast. Levels of each gene are normalised to the lowest expressing cell type and only genes with at least 1.23-fold difference are shown. *P<0.05; **P<0.01; ***P<0.005; ****P<0.0001; one-way ANOVA followed by Tukey's multiple comparisons test.

We confirmed expression of three highly expressed surface proteins on ITGA2^+^ cells: EpCAM, TM4SF1 and CD81. EpCAM (also known as TACSTD1) is expressed by other stem cells and, more importantly, is present specifically on a subset of trophoblast progenitors in the mouse placenta (Ueno et al., 2013). In human placentas, EpCAM is present on VCT and at the base of the columns at 6-7 weeks, but is restricted to the base of the CCCs after 8 weeks (Fig. 5A; *n*=3, G.A.=6-7 weeks; *n*=3, G.A.=8-10 weeks). The expression of TM4SF1 and CD81 is more widespread than EpCAM, but the strongest expression for both surface proteins is at the base of the CCCs (Fig. S3A; *n*=3, G.A.=8-9 weeks). We also identified components of hemidesmosomes, *COL17A1* and *LAMB3*, enriched in ITGA2^+^ cells (Fig. S3B). COL17A1 is expressed mainly at the base of the CCCs and in some EVT (Fig. S3C). The expression of different surface proteins in the proximal CCC will allow further analysis of the heterogeneity within this niche in the future.

Apart from these surface markers, one of the top differentially expressed genes is thymosin beta 4 (TMSB4X), which is associated with the stemness and differentiation of progenitor and cancer cells (Fig. 4D; Table S1) (Bock-Marquette et al., 2009; Lv et al., 2013; Wirsching et al., 2013). TMSB4X increases NOTCH1 activity, and *NOTCH1* is also upregulated in ITGA2^+^ cells (Fig. 4D; Table S1) (Huang et al., 2016; Lv et al., 2013). We therefore investigated the levels of genes involved in NOTCH signalling in the ITGA2^+^ population. *NOTCH1* and its downstream targets *CDKN1A* and *PLAU* are significantly upregulated in ITGA2^+^ cells compared with VCT and EVT, suggesting that NOTCH signalling is active in TPs (Fig. 5B) (ANOVA two-way group analysis, Tukey's multiple comparisons test). *HES2* expression as another target of NOTCH signalling is also enriched in ITGA2^+^ cells with an FDR<0.05. The location of NOTCH1 in human placentas has been controversial (De Falco et al., 2007; Haider et al., 2014; Haider et al., 2016; Herr et al., 2011; Hunkapiller et al., 2011). Our results agree with Haider et al. (2014, 2016) that NOTCH1 is present at the base of the columns and demarcates a progenitor-like population.

Because NOTCH1 is involved in the regulation of epithelial-mesenchymal transition (EMT), and this pathway also came out of our gene set enrichment analyses (Fig. 5C), we looked at the expression of EMT genes amongst the A-E-G populations (Fig. 5D, Fig. S3D) (Wang et al., 2011). EMT genes that are 1.23 times higher in at least one of the populations were selected. Most of the gene expression changes between the EGFR^+^ and HLA-G^+^ populations reflect the epithelial and mesenchymal nature of VCT and EVT, respectively, as previously described (Fig. S3D) (Davies et al., 2016). The ITGA2^+^ subgroup of cells, however, shows an intermediate phenotype between these two extremes. As such, it is more akin to VCT in terms of *CDH1* (cadherin 1; also known as E-cadherin) expression, but at the same time expresses high levels of *TPM1* and *TPM2*, more similar to EVT cells (Fig. S3D). Moreover, we identified a particular group of EMT-related genes that are exclusively upregulated in the ITGA2^+^ population (Fig. 5D).

Taken together, the transcriptome of the A-E-G populations shows that ITGA2 demarcates a distinct population of proliferative cells at the base of the CCCs. To understand more about the differentiation capacity of these cells, we performed lineage tracing on human first trimester placentas.

### Cells in the proximal column can contribute to VCT and EVT

**Fig. 6.**
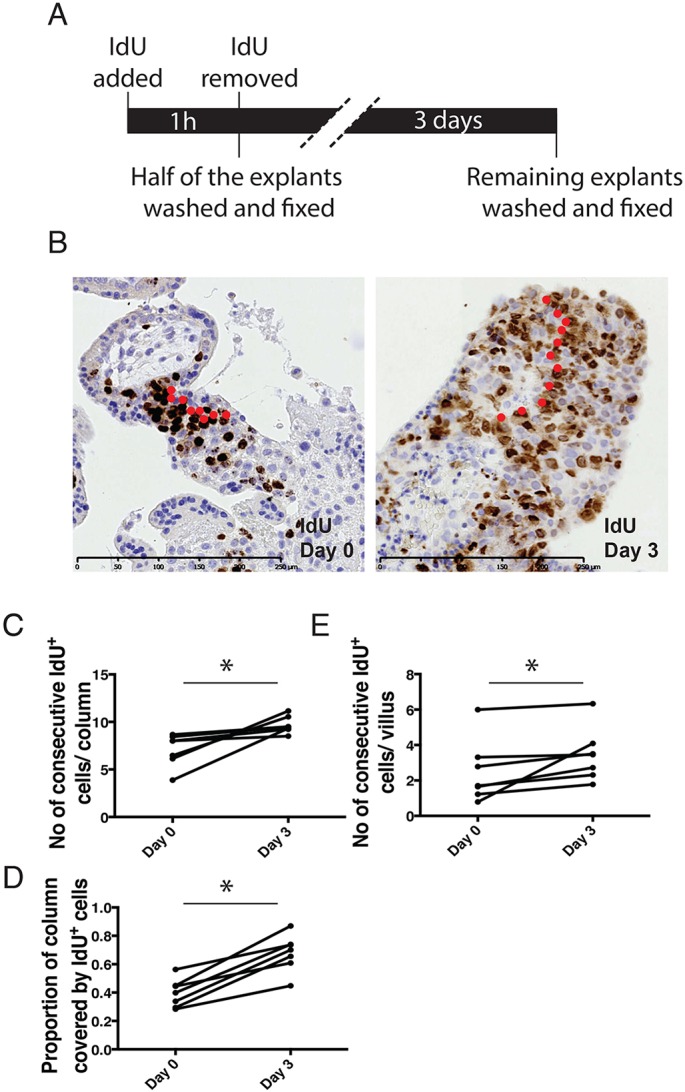
**Cells at the base of the CCCs could contribute to both VCT and EVT.** (A) Time line for lineage tracing. (B) An example of how the longest consecutive IdU^+^ streak is counted in CCCs in Day 0 and Day 3 samples. IdU^+^ cells within the longest string are marked by a red dot. (C-E) There is an increase in the average number of consecutive IdU^+^ cells in the CCCs (C), in the area of IdU^+^ cells in the CCCs (D), and in the number of IdU^+^ VCT in the villi adjacent to CCCs (E) after 3 days (*n*=7). **P<0.01; Mann–Whitney *U*-test.

Because lineage tracing by genetic manipulation is not practicable in human placentas, we used IdU labelling of explants as a proxy hereditary label to trace the fate of cells. Specifically, cells were treated with IdU for 1 h on Day 0, and half of the explants were fixed immediately whereas the other half were fixed after 3 days (Fig. 6A). With IdU portioned equally between daughter progeny following division, the positional fate of cells could be traced over time.

Following short-term IdU incorporation, IdU^+^ cells were visible in a region close to the base of the CCCs as described above, localising high proliferative activity to cells in this niche (Fig. 1B). To trace the fate of these proliferative cells, we then examined the pattern of IdU staining after 3 days of chase. This allowed the migration of IdU-labelled cells into the columns to be assessed in two ways. The first method relied on the tendency of IdU^+^ cells to form ‘strings’ of consecutive positively labelled cells in the proximal CCC. We reasoned that the change in the length of the string could provide an estimate of the cell migration rate from the base region. To control for potential clone variation associated with lateral division, only cells in the longest string in each column were scored. In this way, we obtained an estimate for the average length of the longest string per column for each donor placenta between Day 0 and Day 3. An example of how cells in the string were counted is shown in Fig. 6B. The results show that there is a net increase in string size after 3 days (Fig. 6C; *n*=7, G.A.=8-10 weeks, *P*<0.01, Mann–Whitney *U*-test). To verify that the strings were representative of what is happening in the entire column, we measured the area of the regions containing labelled cells and the area of the entire columns. By comparing the ratio of the area of labelled cells with that of the entire column between Day 0 and 3, we confirmed that there are more labelled cells in the column after 3 days (Fig. 6D; *n*=7, G.A.=8-10 weeks, *P*<0.01, Mann–Whitney *U*-test).

A similar strategy was employed for studying whether the proliferative trophoblast in the proximal CCC can contribute to the villus. As there tend to be consecutive IdU^+^ VCT cells at the edge of the CCCs, the average number of consecutive positive VCT cells was compared between Day 0 and Day 3. There was a net gain in the number of consecutive IdU^+^ VCT cells near the edge of the columns (Fig. 6E; *n*=7, G.A.=8-10 weeks, *P*<0.01, Mann–Whitney *U*-test). This suggests that the proliferative cells at the base of the CCCs could contribute to both villi and columns.

## DISCUSSION

The identity and location of TP cells in early human placentas are still unknown. We have identified ITGA2 as a surface marker on a subpopulation of proliferative trophoblast residing at the base of the CCCs that can probably contribute to both VCT and EVT. Using it, we have been able to isolate and characterise putative TPs. We used placentas from the first trimester to study TPs, as expression of proliferative markers decreases with gestational age in the human placenta (Arnholdt et al., 1991; Hemberger et al., 2010; Horii et al., 2016). Similarly, EpCAM is expressed in VCT early in the first trimester but becomes restricted to the proximal CCC at approximately 8 weeks. This evidence indicates that the proliferative niche becomes more restricted as gestation proceeds, in keeping with the exponential growth of the placenta very early in gestation. This is analogous to the gut where proliferation is initially present throughout the epithelium but becomes confined to the crypts during development (Noah et al., 2011).

Sites of proliferation are commonly associated with progenitor cells. To locate these in early pregnancy, we used both Ki67 and IdU on sections of first trimester placentas. In agreement with others, we found that proliferative markers are concentrated at the base of the CCCs, with less proliferation occurring in the VCT population scattered around the villi between 7-11 weeks (Arnholdt et al., 1991; Bulmer et al., 1988; Hemberger et al., 2010; Korgun et al., 2006; Lash et al., 2006; Mühlhauser et al., 1993; Westerman et al., 2004). Our lineage tracing results suggest that these cells localised at the base of the CCCs may be able to contribute to both VCT and EVT. Next, because the stem/progenitor cell niche is often characterised by the expression of particular integrin subtypes in a range of tissues, we looked for an integrin for which expression was restricted to the base of the CCCs (Chen et al., 2013). We found that ITGA2 marks a small group of cells in this proliferative niche and can be used to isolate the putative TPs. Villous endothelial cells also express ITGA2 but these can be excluded using the specific endothelial marker CD34. ITGA2 can only form heterodimers with ITGB1, and integrin α2β1 binds to collagen I, II, IV, XI and XXIII as well as laminin (Tuckwell et al., 1995, 1996; Tulla et al., 2001; Veit et al., 2011). Although collagen I was upregulated in ITGA2^+^ trophoblast in our microarray analysis, we found significant protein expression only in the villous stromal core and not in the CCCs (data not shown), thus making a functional interaction of ITGA2 with one of the other binding partners more likely. Mice lacking the *Itga2* gene are viable and exhibit no placental defects, suggesting that it is not essential for murine trophoblast development (Chen et al., 2002). Although the role of ITGA2 in trophoblast cells is unclear, it also marks proliferating cells in other organs (Beaulieu, 1992; Lussier et al., 2000; Zutter and Santoro, 1990).

We used flow cytometric cell sorting to isolate the ITGA2^+^ cells at the base of the CCCs and performed a transcriptome analysis to identify their specific expression profile. Amongst the upregulated genes in the ITGA2^+^ trophoblast, we identified *COL17A1* and *LAMB3*, components of hemidesmosomes. COL17A1 is crucial for the maintenance of hair follicle stem cells and deficiency of COL17A1 leads to loss of the stem cell signature and premature hair ageing (Tanimura et al., 2011). Moreover, the product of *SFN* (stratifin or 14-3-3σ), another ITGA2^+^ upregulated gene, can bind both COL17A1 and keratins, thereby bridging the hemidesmosomes to the rest of the cytoskeleton (Li et al., 2007). Indeed, the balance between proliferation and differentiation in skin has parallels with the CCCs and differentiation to EVT.

We also find that ITGA2^+^ trophoblast cells are enriched in genes involved in the NOTCH1 signalling pathway, *HES2*, *CDKN1A*, *PLAU* and *MYC*. Moreover, TMSB4X as an ITGA2^+^-enriched factor is known to increase the expression of *NOTCH1*, providing evidence for the potentially self-reinforcing nature of the ITGA2 niche (Huang et al., 2016; Lv et al., 2013). As expression of different NOTCH receptors is restricted to subsets of trophoblast cells, and the inhibition of NOTCH signalling elicits two opposing effects of proliferation and differentiation in a mixed population of trophoblast cells, it will be interesting to study the specific inhibition of NOTCH1 in ITGA2^+^ trophoblast *in vitro* (Haider et al., 2014, 2016).

As cells in the CCCs move away from the basement membrane and invade the decidua upon attachment to the uterus, they undergo a process typical of EMT (Davies et al., 2016). We find that ITGA2^+^ trophoblast cells at the base of the CCCs exhibit an unusual expression repertoire of both epithelial and mesenchymal markers. Even within the group of genes generically classified as ‘mesenchymal’, most of them are not expressed in EVT. These data suggest that the ITGA2^+^ subpopulation is in a unique state between epithelial-like VCT and the more mesenchymal-like EVT. This phenotype is in line with the position of ITGA2^+^ cells at the interface between both groups, supporting the notion that they may be capable of contributing to both of these major trophoblast populations.

The flow cytometry data further corroborate this point as the ITGA2^+^ population is proliferative and contains both EGFR^+^ VCT and HLA-G^+^ EVT cells. The truly bipotential capacity of these cells is difficult to prove, however, as neither lineage tracing of human trophoblast nor live imaging are possible because of the optical density of the CCCs and the floating nature of the villi. We used a thymidine analogue pulse-chase strategy as a preliminary attempt to study the differentiation potential of the cells in this proliferative niche. Based on the contribution of IdU^+^ cells, we conclude that the cells at the base of the CCCs could contribute to both VCT and EVT, although we cannot rule out the presence of separate progenitor cells with different lineage contributions.

Taken together, we have identified a cell surface marker, ITGA2, which marks a proliferative TP compartment in the first trimester placenta that is regulated by NOTCH signalling and exhibits unique expression characteristics. These insights will help elucidate the putative stem or progenitor cell niche in the early human placenta for future attempts at culturing a self-renewing human trophoblast cell population.

## MATERIALS AND METHODS

### Ethics

Ethical approval was obtained from the Cambridgeshire 2 Research Ethics Committee (reference no. 04/Q0108/23; Cambridge, United Kingdom) and informed written consent was obtained from each patient.

### Immunohistochemistry and confocal microscopy

Frozen sections were used for immunohistochemistry except for anti-IdU and anti-COL17A1, for which formalin-fixed, paraffin-embedded sections were used. Pieces of placenta were embedded in OCT, snap-frozen in liquid nitrogen and sectioned at 5 µm thickness, then fixed in acetone. Paraffin sections were dewaxed and placed at 125°C for 30 s and then 90°C for 10 s in citrate buffer (Menarini Diagnostics). For colorimetric staining, the sections were blocked in 2.5% horse serum, and then incubated in primary antibody (see Table S3 for details), biotinylated secondary antibody (Vectastain, #BA-2000, #BA-9500, BA-1100) and HRP-conjugated ABC complex (Vectastain, #PK-6100). Each incubation was 30 min long, with two 5 min washes in 0.1% Tween/PBS between each incubation. Incubation for anti-COL17A1 was overnight at 4°C. The sections were then developed with 3,3′-diaminobenzidine (DAB) (Sigma, #D4168). For co-immunofluorescence staining, the sections were incubated in primary antibody overnight at 4°C and then in fluorophore-conjugated secondary antibody (Invitrogen, #A-11029, #A-11011, 1:400) for 2 h. Coverslips were mounted using Vectashield mounting medium containing DAPI (#H1200) and images were acquired with a Zeiss LSM 700 confocal microscope.

### Isolation of cells from human placentas

To obtain single cells from the placenta, the chorionic villi were scraped off from the membranes and incubated in 0.2% trypsin at 37°C for 9 min. The resulting mixture was sieved through muslin cloth and the flow through was centrifuged at 400 ***g***. The pellet was re-suspended in 8 ml of Ham's F12 medium, filtered through a 100 µm cell sieve, then layered onto 8 ml of Lymphoprep (Axis-shield, #1114544) and centrifuged at 700 ***g*** for 20 min at room temperature. Mononuclear cells were recovered from the interphase between the Lymphoprep and Ham's F12 medium.

### Staining for surface and intracellular proteins for flow cytometry

Live cells were blocked with 0.25 mg/ml human immunoglobulin (Sigma, #I4506), followed by incubation in fluorophore-conjugated antibodies (see Table S4 for details) for 30 min at 4°C. They were then washed in 1% fetal calf serum (FCS) in PBS and fixed in 2% paraformaldehyde (PFA). For cell sorting, cells were not fixed and were sorted by Cytomation MoFlo immediately.

To stain for KRT7 and Ki67, cells were fixed in Foxp3 fixation/permeabilisation reagent (eBioscience, #00-5521-00) for 30 min in the dark after staining for surface proteins and permeabilised in eBioscience Permeabilisation Buffer (#00-8333). They were then blocked in human immunoglobulin and incubated with fluorophore-conjugated antibodies (see Table S4 for details) for 15 min at room temperature. They were subsequently washed in eBioscience Permeabilisation Buffer and fixed in 2% PFA.

The data acquisition was performed using either Cytek Development DxP 8 colours (488/637/561) or BD LSRFortessa (405/488/637/561). All compensation was applied digitally post-acquisition. The data were analysed using FlowJo (Tree Star).

### Extraction of RNA

Cells were lysed in TRIzol Reagent (Invitrogen, #15596026) and 200 µl of chloroform was added for every 1 ml of TRIzol. The mixture was then vortexed and centrifuged at 12,000 ***g***, 4°C, for 15 min. The upper aqueous phase was transferred to a new tube, 1.5 times volume of ethanol was added and the entire content was then centrifuged through Purelink columns (Invitrogen, #12183-016). RNA was purified from the columns following the manufacturer's protocol, including the DNase step. The final product was eluted in 12 µl of RNase-free water.

### RT-qPCR

RNA was converted into cDNA using the Transcriptor First Strand cDNA Synthesis Kit from Roche (#04379012001). For each test, 10 µl of 2× Fast SYBR Green Mastermix (Applied Bioscience, #4385612) and 10 ng of cDNA were used, and each gene was tested in triplicate using the Applied Biosystems 7500 Real-Time PCR System. The settings of the machine were: 95°C 5 min, then 40× (95°C 10 s, 60°C 30 s). Melting curve analysis was carried out at the end, to ensure that there was only one amplicon. All genes were normalised to *TBP* levels. See Table S5 for primer sequences.

### Microarray

Four donor sets of three cell types with RNA integrity number (RIN) above 7 as measured by 2100 Bioanalyzer Instrument (Agilent Technologies) were amplified using the Ovation Pico WTA System v2 kit (NuGEN, #3302) according to the manufacturer's protocol. Good quality final products as assessed by the 2100 Bioanalyzer Instrument were biotinylated using the Encore Biotin Module by NuGEN (#4200-12) and hybridised to Illumina Human HT-12 V4 BeadArray (#BD-103-0204) using the manufacturer's protocol. After correcting for background, the output data from Illumina BeadStudio were logged to the base of two and multiple testing corrections were applied to paired-wise analysis of each cell type against each other in the R statistical program. The data were filtered to remove probes for which the detection *P*-value was not above 0.01 for at least one sample for the analysis.

Microarray work and analysis was performed by Cambridge Genomic Services. Gene functional annotation analysis was carried out using Database for Annotation, Visualization and Integrated Discovery (DAVID) (Huang et al., 2009a,b) and Gene Set Enrichment Analysis (GSEA) (Subramanian et al., 2005; Mootha et al., 2003).

### Bisulphite sequencing

DNA was extracted from TRIzol-lysed samples after the RNA had been removed from the aqueous phase, according to the manufacturer's protocol. For bisulphite conversion, 10 ng of DNA was treated using the EpiTect Bisulfite Kit (Qiagen, #59110), according to the manufacturer's protocol. The region upstream of *ELF5* transcription start site was then amplified by nested PCR. See Table S6 for primer sequences.

Amplicons were purified from agarose gel using QIAquick Gel Extraction Kit (Qiagen, #28704) and cloned into the pGEM-T Easy vector (Promega, #A137A). The ligations were transformed into Library Efficiency DH5_α_ Chemically Competent Cells (Invitrogen, #18263012) and eight colonies per group were selected to be sequenced; these were confirmed as representing distinct alleles.

### Lineage tracing by pulse chasing

Samples from seven donors were cut into roughly 10×10×10 mm pieces, avoiding pieces nearer to the edge of the placenta. During IdU incubation, they were suspended in 10% FCS/DMEM±80 µM IdU (Alfa Aesar, #A11542), with three pieces of explants/well in a 6-well plate coated with 1% gelatin (1 ml/well). At least three random explants per donor were collected at each time point. The explants were fixed in 4% PFA overnight, switched to 70% ethanol for storage, and embedded in paraffin for sectioning. For antigen retrieval and DNA denaturation, sections were immersed in Access Super RTU buffer (pH 9; Menarini Diagnostics) and placed in a Menarini Access Retrieval Unit and subjected to high pressure at 125°C for 1 min and then 90°C for 10 s. The slides were then stained as per normal.

### Statistics

All error bars on graphs represent s.e.m. Statistical tests used are indicated for each figure.

## Supplementary Material

Supplementary information
