# Supplementary material for: Integrin α2 marks a niche of trophoblast progenitor cells in first trimester human placenta
Source: Development. 2018 Apr 16;145(16):dev162305. doi: 10.1242/dev.162305 (PMC6124543; doi:10.1242/dev.162305)
Supplement: Supplementary information [file develop-145-162305-s1.pdf]

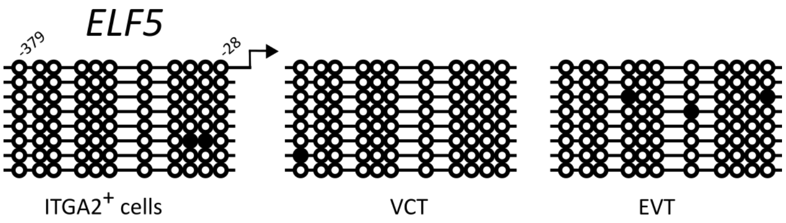

Figure S1. The methylation profile of the *ELF5* promoter in ITGA2^{+} cells, VCT and EVT. Open circle = unmethylated CpG; closed circle = methylated CpG.

Development • Supplementary information

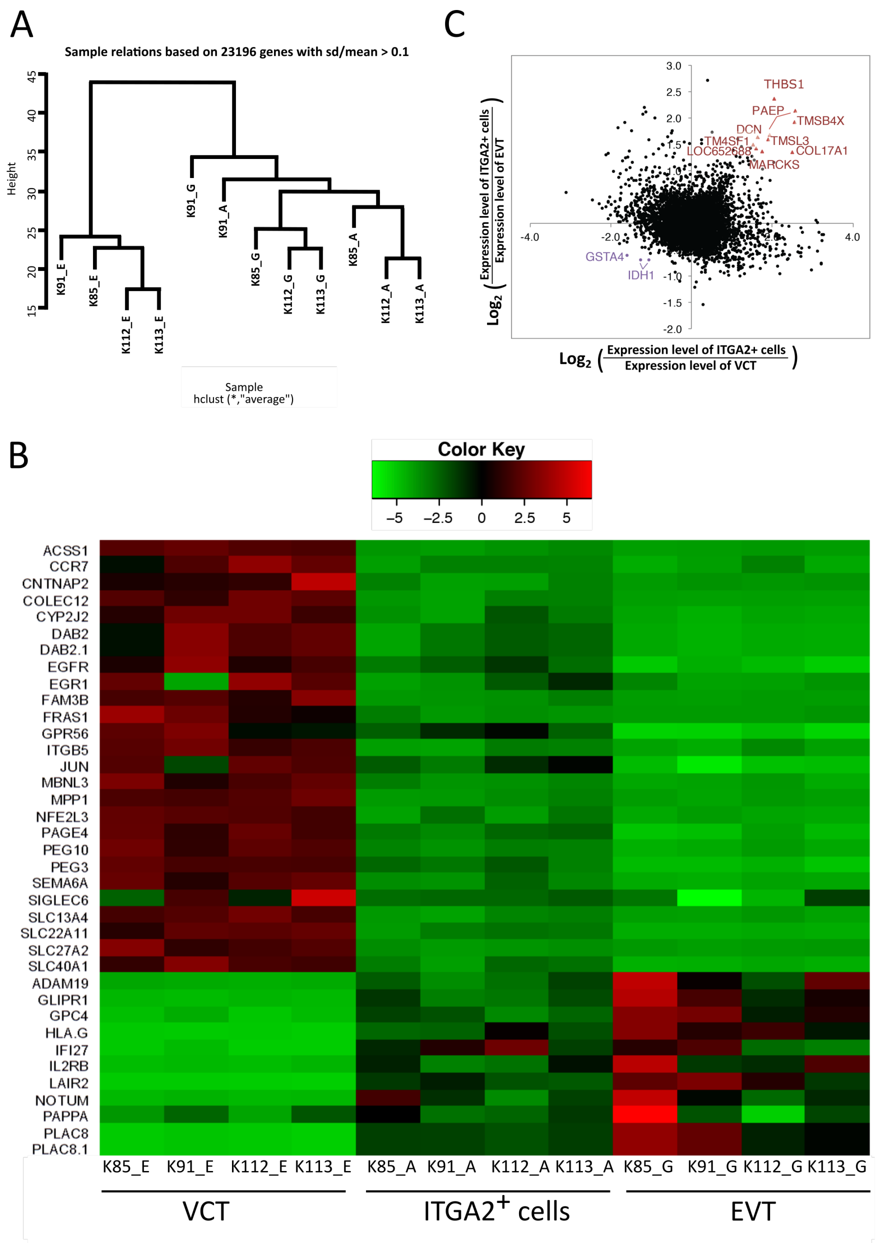

Figure S2. Validation of the microarray. (A) Hierarchical clustering of the A-E-G populations. (B) Highly upregulated genes in VCT and EVT based on a previous microarray are also highly expressed in these cells on our microarray (Apps et al., 2011). (C) Overview of the relative expression levels between ITGA2^{+} trophoblast compared to those in VCT and EVT. There are very few genes that are lower in ITGA2^{+} trophoblast than in VCT and EVT.

Development • Supplementary information

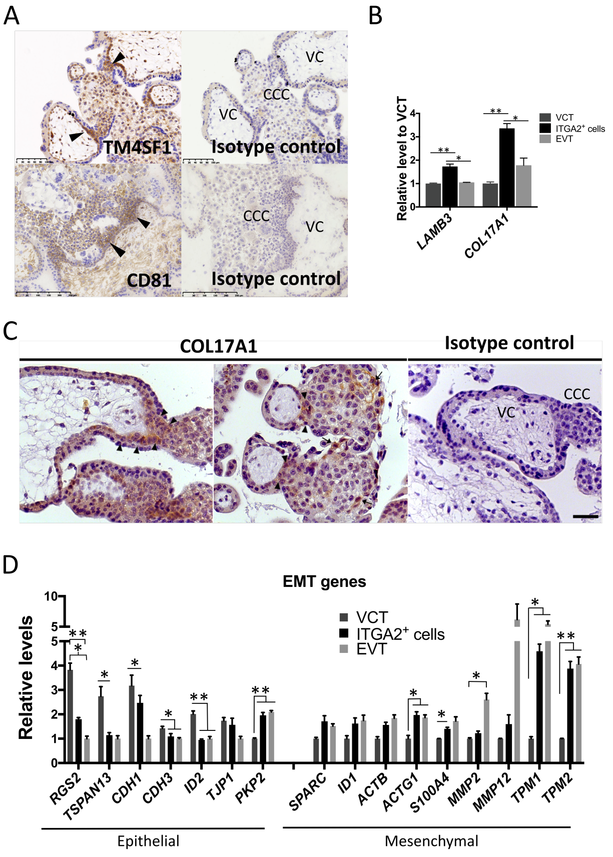

Figure S3. Expression profile of EMT genes, CD81 and TM4SF1. (A) CD81 and TM4SF1 are upregulated at the base of the CCCs (arrow heads). VC = villous core; CCC = cytotrophoblast cell column. (B) Expression of *LAMB3* and *COL17A1* on the microarray. Stats for individual genes: * = $p < 0.05$; ** = $p < 0.01$; *** = $p < 0.005$; **** = $p < 0.0001$; One-way ANOVA followed by Tukey's multiple comparisons test. (C) COL17A1 is upregulated at the base of the columns (arrowheads) and in some EVT (arrows). Scale bar = 50 μm. VC = villous core; CCC = cytotrophoblast cell column. (D) The expression of epithelial and mesenchymal genes is higher in VCT and EVT respectively, based on the microarray. The following genes were either not detected or not significantly different between all groups and were not included in the graph: *SNAI1, SNAI2, TWIST1, TCF, ACTA2, LEF1, ZEBs, CDH3, CRB3, OCLN, TJP1, Claudins, DSP, SPARC, ID1, CFL1 and other MMPs.*

Development • Supplementary information

Table S1. Genes upregulated in ITGA2^{+} cells, compared to VCT and EVT, on microarray. FDR <0.05, Fold change ≥1.23

Click here to Download Table S1

Table S2. Genes downregulated in ITGA2^{+} cells, compared to VCT and EVT, on microarray. FDR <0.05, Fold change ≥1.23

**Symbol**	**Fold decrease in ITGA2^{+} cells compared to EVT**	**Fold decrease in ITGA2^{+} cells compared to VCT**
GSTA4	1.53	3.00
IDH1	1.61	2.07
HIBADH	1.28	1.55
ATAD1	1.31	1.48
FAM89A	1.44	1.47
LIPA	1.50	1.44
ADAP2	1.36	1.42
POP5	1.29	1.40
RSU1	1.33	1.40
POLR3B	1.40	1.38
PPHLN1	1.27	1.37
FLAD1	1.37	1.35
GXYLT1	1.31	1.34
PAQR3	1.36	1.34
CYFIP2	1.65	1.32

Table S3. Antibodies used for IHC and co-immunofluorescence

Antigen	Brand	Catalogue number	Clone	Dilution factor
ITGA2	R&D	MAB1233	HAS3	1/200
TFAP2C	R&D	AF5059	–	1/100
IdU	BD Biosciences	347580	B44	1/50
GATA3	R&D	AF2605	–	1/100
Ki67	A Menarini	MP-325-CRM01	SP6	1/100
TM4SF1	Sigma Aldrich	HPA002823	–	1/350
CD34	Dako	M716529-2	QBEnd10	1/100
KRT7	Dako	M701829-2	OVTL12/30	1/200
COL17A1	Abcam	Ab186415	EPR14758	1/100
EpCAM	BD Biosciences	347197	EBA-1	3/100
CD31	Dako	M082329-2	JC70A	1/100
CD81	BD Biosciences	561957	JS-81	3/100

Table S4. Antibodies used for flow cytometry

Antigens	Brand	Catalogue number	Clone	Fluorophore	Dilution factor
ITGA2	R&D	FAB1233P	HAS3	PE	1/100
HLA-G	Lab-produced	–	G233	Pacific blue	1/200
Ki67	BD Biosciences	556026	MOPC-21	FITC	3/100
KRT7	Millipore	CBL194F	LP5K	FITC	1/100
CD45	R&D	FAB1430A	2D1	APC	3/40
CD34	BD Biosciences	555824	581	APC	1/100
EGFR	AbD Serotec	MCA1784F	ICR10	FITC	1/20

Development • Supplementary information

Table S5. Primers used for RT-qPCR

Gene	Forward primer (5' to 3')	Reverse primer (5' to 3')
CDH1	GAACGCATTGCCACATACAC	ATTCGGGCTTGTTGTCATTC
EMP3	CGAGAATGGCTGGCTGAAG	GCCACGCTGGTGCAAAG
ITGA2	TCACCAGGAACATGGGAACT	GTCAGAACACACACCCGTTG
ITGB6	CTACCTGTGGTGACCCCTGTAAC	GCTTGGCCAGCTGCTGAC
LAIR2	CACACCTCACTGCTCTCCTG	TGAAAGTCACATGGCTCCCC
RARRES3	TGGGCCCTGTATATAGGAGATG	GGACTGAGAAGACACTGGAGGA
SOX13	AAGGATGAGCGGAGGAAGAT	GACTTCCAGCGAGATCCAAG
TBP	GAGCTGTGATGTGAAGTTTCC	TCTGGGTTTGATCATTCTGTAG

Table S6. Primers used for bisulphite sequencing

Primer name	Sequence
hELF5-2b BiS -483F	GGAAATGATGGATATTGAATTTGA
hELF5-2b BiS +31R	CAATAAAAATAAAAACACCTATAACC
hELF5-2b BiS -432F	GAGGTTTTAATATTGGGTTTATAATG
hELF5-2b BiS -3R	ATAAATAACACCTACAAACAAATCC

Development • Supplementary information
